# Impact of telemetry supported home blood pressure monitoring: experiences of patients and professionals participating in the HITS randomised controlled trial of telemetry enabled home blood pressure (BP)

**Published:** 2012-06-15

**Authors:** Janet Hanley, Jenny Ure, Mary Paterson, Sarah Wild, Paul Padfield, Claudia Pagliari, Brian McKinstry

**Affiliations:** Edinburgh Napier University, UK; University of Edinburgh, UK; University of Edinburgh, UK; University of Edinburgh, UK; Scottish Government Health Department, UK; University of Edinburgh, UK; University of Edinburgh, UK

**Keywords:** blood pressure monitoring, primary care

## Abstract

**Aim:**

To explore the experiences of participants in a randomised controlled trial of telemetry supported home blood pressure monitoring for people with hypertension in order to explain the outcomes and guide further service development.

**Method:**

Twenty-five patients and the healthcare teams from 6 (of 20) participating practices were interviewed. Transcribed interviews were analysed thematically with verification through partial double coding, team review, and presentation of the analysis and sections of coded data back to participants.

**Results:**

Doctors, nurses and patients were aware that BP measured in the surgery could be a poor indicator of usual BP and this delayed changes to treatment. The rolling average blood pressure from telemetry enabled home monitoring, visible to both the patient and practice, was accepted as a good indicator which should prompt changes in self care and treatment. Although patients had different emotional reactions to home monitoring (some saying that focusing on a health risk they normally did not think about increased anxiety, most saying that home monitoring reassured them), many provided examples of using the data to monitor their response to self care changes such as increasing exercise, and to medication changes. They also used the data in healthcare consultations to be more explicit in negotiating what they wanted. Healthcare professionals mostly considered home monitoring to be empowering for patients, but shared the concern that for a few patients it may increase anxiety. Managing telehealth was challenging to practice organisation and individual work patterns, and increased patient contacts with the practice. This was difficult to sustain using traditional face-to-face contacts and there was increased use of telephone contacts and some experimentation with email. Some professionals questioned the value of reaching the target BP on overall cardiovascular risk and reluctance to increase medication and resistance from patients was a theme, although not a major one.

**Conclusion:**

Telemetry enabled home BP measurement has a positive effect on the management of hypertension. The main enabling factor was increased trust in a measurement taken at home and based on multiple readings. However, it was not easy for professionals to incorporate telehealth in their usual working practices.

